# Adrenomedullin Is a Diagnostic and Prognostic Biomarker for Acute Intracerebral Hemorrhage

**DOI:** 10.3390/cimb43010027

**Published:** 2021-06-11

**Authors:** Francisco J. Julián-Villaverde, Laura Ochoa-Callejero, Eva Siles, Esther Martínez-Lara, Alfredo Martínez

**Affiliations:** 1 Stroke Unit, Neurology Service, Hospital San Pedro, 26006 Logroño, Spain; fjulian@riojasalud.es; 2 Angiogenesis Group, Oncology Area, Center for Biomedical Research of La Rioja (CIBIR), 26006 Logroño, Spain; locallejero@riojasalud.es; 3 Experimental Biology Department, University of Jaén, 23071 Jaén, Spain; esiles@ujaen.es (E.S.); elara@ujaen.es (E.M.-L.)

**Keywords:** adrenomedullin, acute hemorrhagic stroke, circulating levels, diagnostic biomarker, prognostic biomarker

## Abstract

Hemorrhagic stroke remains an important health challenge. Adrenomedullin (AM) is a vasoactive peptide with an important role in cardiovascular diseases, including stroke. Serum AM and nitrate–nitrite and S-nitroso compounds (NOx) levels were measured and compared between healthy volunteers (*n* = 50) and acute hemorrhagic stroke patients (*n* = 64). Blood samples were taken at admission (d0), 24 h later (d1), and after 7 days or at the time of hospital discharge (d7). Neurological severity (NIHSS) and functional prognosis (mRankin) were measured as clinical outcomes. AM levels were higher in stroke patients at all times when compared with healthy controls (*p* < 0.0001). A receiving operating characteristic curve analysis identified that AM levels at admission > 69.0 pg/mL had a great value as a diagnostic biomarker (area under the curve = 0.89, sensitivity = 80.0%, specificity = 100%). Furthermore, patients with a favorable outcome (NIHSS ≤ 3; mRankin ≤ 2) experienced an increase in AM levels from d0 to d1, and a decrease from d1 to d7, whereas patients with unfavorable outcome had no significant changes over time. NOx levels were lower in patients at d0 (*p* = 0.04) and d1 (*p* < 0.001) than in healthy controls. In conclusion, AM levels may constitute a new diagnostic and prognostic biomarker for this disease, and identify AM as a positive mediator for hemorrhagic stroke resolution.

## 1. Introduction

Stroke, a cerebrovascular accident, is the second major cause of morbidity and mortality in many developed countries and its incidence is increasing because of population ageing [1]. In fact, the global lifetime risk of stroke in adults over the age of 25 is approximately 25% [2], with 24.9 million cases of ischemic stroke, and 18.7 million cases of hemorrhagic stroke reported in 2015 [3]. Ischemic stroke is more frequent but hemorrhagic stroke is responsible for more deaths and disability-adjusted life-years lost [4]. Spontaneous hemorrhagic stroke is due to bleeding into the brain by the rupture of a blood vessel without a cerebral vascular malformation. Depending on the localization of the bleeding, it is further subdivided into deep supra or infratentorial intracerebral hemorrhage (mostly secondary to hypertension small-vessel disease) or cortical hemorrhage (mostly related to cerebral amyloid angiopathy). Others causes of hemorrhagic stroke are tumor related, drugs (e.g., cocaine), trauma, and other uncommon entities (e.g., endocarditis, angiitis, dissections) [5].

Non-modifiable risk factors for hemorrhagic stroke include age, sex, and race/ethnicity, whereas modifiable factors encompass hypertension, smoking, diet, and physical inactivity, among others [6]. Some genetic risk factors have been identified for hemorrhagic stroke, including some monogenic causes [7] and some sex-specific loci [8].

Blood pressure management, coagulation reversal, neurosurgical treatment, and intracranial pressure control are the mainstays of hemorrhagic stroke treatment [9]. Furthermore, specific rehabilitation protocols help in patient recovery [10], and new experimental approaches including stem cell therapy [11] and nanomedicine [12] are being developed.

One of the main challenges in developing effective new therapeutics and long-term interventions for stroke recovery is the heterogeneity of stroke, including etiology, comorbidities, and lifestyle factors that affect every stroke survivor. In this regard, identifying biomarkers of stroke is an active area of research since their potential use is not limited to diagnosis, but includes also prognosis and patient monitoring [13]. In the case of hemorrhagic stroke, a fast and accurate diagnosis is crucial for the immediate application of the right therapy, and the identification of blood biomarkers may help to distinguish stroke from stroke mimics, and ischemic from hemorrhagic stroke [14].

Adrenomedullin (AM) is a vasodilator peptide produced by numerous areas of the central nervous system [15] and peripheral tissues [16]. AM expression is upregulated under hypoxia via activation of the hypoxia inducible factor-1 (HIF-1) pathway [17]. AM levels constitute an independent predictor of future cardiovascular events [18] and they have been shown to increase following ischemic insults to the brain [19,20] and traumatic brain injuries [21,22]. Previous studies in mice lacking AM expression suggest that this peptide is neuroprotective in the context of ischemic stroke, and may exert its effects via regulation of nitric oxide synthase (NOS), matrix metalloproteinases, and inflammatory mediators [23]. The levels of circulating AM are elevated in ischemic stroke patients and may predict patient outcome [24,25]. Regarding hemorrhagic stroke, AM blood levels were reported in a single study on intracerebral hemorrhage [26] and another on subarachnoid hemorrhage [27]. In addition, changes of AM on the cerebrospinal fluid were found in subarachnoid hemorrhage patients [28,29]. All these studies reported elevated levels of AM when comparing hemorrhagic stroke patients with the healthy population, but the evolution of those levels during disease progression was not studied.

The goal of this study was to determine the serum levels of AM in patients with acute intracerebral hemorrhage at different times and compare them with a group of healthy volunteers, and to determine the potential value of these levels as a diagnostic or prognostic biomarker.

## 2. Materials and Methods

### 2.1. Ethical Issues

All procedures were approved by the review boards of La Rioja (Comité de Ética de Investigación con medicamentos de La Rioja, CEImLar, ref. PI-319) and Jaén (Comisión de Ética de la Universidad de Jaén, ref. OMGsABs-2268). All described procedures adhere to the tenets of the Declaration of Helsinki.

### 2.2. Patients and Volunteers

The study was designed as a prospective, observational, and longitudinal clinical study with patients diagnosed with acute intracerebral hemorrhage at the Neurology Service of Hospital San Pedro (Logroño, Spain) from December 2018 to January 2020. Consecutive patients fulfilling inclusion criteria signed the informed consent documents and were recruited into the study. Inclusion criteria were patients with intracerebral hemorrhage demonstrated on computed tomography (CT) scan with less than 6 h from onset of symptoms. Exclusion criteria included isolated subarachnoid hemorrhage, cancer related hemorrhage, traumatic intracranial hemorrhage, patients fulfilling organ donation protocol, or neurosurgical hemorrhage.

In this time period, 71 patients arrived to the hospital with intracerebral hemorrhage, but 7 of them fulfilled at least one of the exclusion criteria: CT > 6 h from onset of symptoms (*n* = 1), traumatic intracranial hemorrhage (*n* = 2), cancer related hemorrhage (*n* = 1), organ donation protocol (*n* = 1), and others (*n* = 2). Finally, 64 patients were included in the study.

In addition, 50 sex-matched healthy volunteers were recruited from habitual blood donors at the Blood Bank of the Hospital.

### 2.3. Variables of the Study

Patients received the standard care following the approved protocols of the Neurology Service. General characteristics of the patients were collected as part of the clinical history (age, sex, risk factors, current medical treatment, previous functional situation, etc.). During their stay at the Neurology Service several parameters were continuously monitored (electrocardiogram, systolic and diastolic blood pressure, temperature, and oxygen saturation). Neurological severity was measured with the NIHSS scale [30] at day 0, day 1, and day 7. Functional prognosis was also evaluated with the mRankin scale at 3 months [31]. In addition, blood serum samples were taken just after admission (d0), at day 1 (d1), and at day 7 (d7), to quantify circulating levels of AM. In some patients (*n* = 11), the third sample was taken between days 3 and 5, instead of at day 7, due to early hospital discharge. Hematoma volume evolution was established by comparing the images taken by CT on admission and day 1. Hematoma volume was measured using the ABC/2 formula [32] and hematoma growth was defined by a combined cutoff of 33% relative or 12.5 mL absolute volume increase [33].

### 2.4. Determination of AM Levels

The concentration of AM present in blood serum was determined using a commercially available radioimmunoassay (RIA) kit (Phoenix Europe GmbH, Karlsruhe, Germany). Samples (1 mL) were initially diluted in an equal volume of 0.1% alkali-treated casein in phosphate-buffered saline at pH 7.4, and applied to pre-washed reverse-phase Sep-Pak C-18 cartridges (Waters Corporation, Milford, MA, USA) to remove the AM-binding protein, complement factor H [34]. The peptide fraction was eluted from the C-18 matrix with 3 mL 80% isopropanol containing 0.125 N HCl and freeze-dried overnight, as previously described [25,35]. AM levels contained in lyophilized extracts were then determined by RIA following the manufacturer’s protocols.

### 2.5. Determination of NOx Levels

Nitric oxide (NO) production was indirectly quantified by determining nitrate–nitrite and S-nitroso compounds (NOx), using an ozone chemiluminescence-based assay adapted to plasma samples [25]. In brief, plasma samples were deproteinized with 0.8 N NaOH and 16% ZnSO_4_ solutions (1/0.5/0.5, *w*/*v*/*v*). After centrifugation at 10,000× *g* for 5 min, the resulting supernatants were removed for chemiluminescence analysis in a NO analyzer (NOATM 280i Sievers Instruments, Boulder, CO, USA). NOx concentration was calculated by comparison with standard solutions of sodium nitrate. Final NOx values were expressed as µM.

### 2.6. Statistical Analysis

All statistical analyses were performed with STATA, Rcommander, and SPSS v.21software packages. First, a descriptive analysis of all variables was performed. Categorical variables were expressed as absolute and relative frequencies. Since most of the datasets did not follow a normal distribution (as tested by the Shapiro-Wilk test), continuous variables were defined by the median and interquartile range. Bivariate analyses were performed with Pearson’s χ2 test, modified by Fisher’s exact test, or in the case of continuous variables with non-parametric tests such as Kruskal-Wallis followed by Mann-Whitney’s U test. The optimal AM cut-off point to differentiate between stroke patients and healthy controls was determined by Youden’s J statistic [36].

## 3. Results

The study group included 64 acute intracerebral hemorrhage patients, 26 women (40.6%) and 38 men (59.4%), with a median age of 81 years (Table 1). Some patients had relevant risk factors, such as arterial hypertension, diabetes, dyslipidemia, or atrial fibrillation. An important proportion of these patients had been treated with antihypertensives, statins, anti-aggregants, or anticoagulants (Table 1). After completing the etiologic profile, more than half of the patients (64.1%) were diagnosed with supratentorial stroke, 21.8% with lobar stroke, 9.4% with infratentorial stroke, and 4.7% with mixed stroke (Table 1). The median NIHSS taken at admission was 7.5 with a median hematoma size of 4.5 cm^3^ (Table 1). Several patients (15/64, 23.4%) died in the 3 months following stroke onset. Healthy volunteers had the same sex frequency but were significantly younger than the stroke patients (Table 1).

### 3.1. Serum AM Levels Are Higher in Intracerebral Hemorrhage Patients Than in Healthy Controls

**Figure 1:**
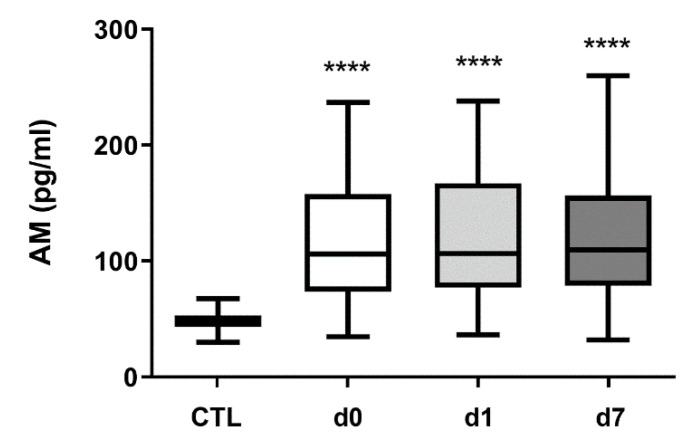
Circulating AM levels increase in hemorrhagic stroke patients. AM levels were measured by RIA in 50 healthy volunteers (CTL) and in 64 hemorrhagic stroke patients. In patients, blood samples were taken on admission (d0), 24 h later (d1), and at hospital discharge (d7). Box plots represent the interquartile range with the median as a horizontal line. Whiskers encompass the maximum and minimum values of the population. ****: *p* < 0.0001, compared to CTL.

In healthy control subjects, AM levels (median, Q1-Q3) were 47.7 pg/mL (43.3–52.8) (Figure 1). In hemorrhagic stroke patients, AM levels were 105.8 pg/mL (74.6–157.8) (d0), 106.4 pg/mL (79.3–166.4) (d1), and 109.7 pg/mL (79.2–154.4) (d7), all of them very significantly higher (*p* < 0.0001), about a 2.5-fold increase, than those obtained in healthy subjects (Figure 1). No differences in AM levels were observed between sexes (*p* = 0.11). Since the age of the patients did not coincide with that of the healthy volunteers, we investigated whether there was a correlation between AM levels and age. A Spearman’s analysis indicated that there is no significant correlation (r = −0.21, *p* = 0.09).

**Figure 2:**
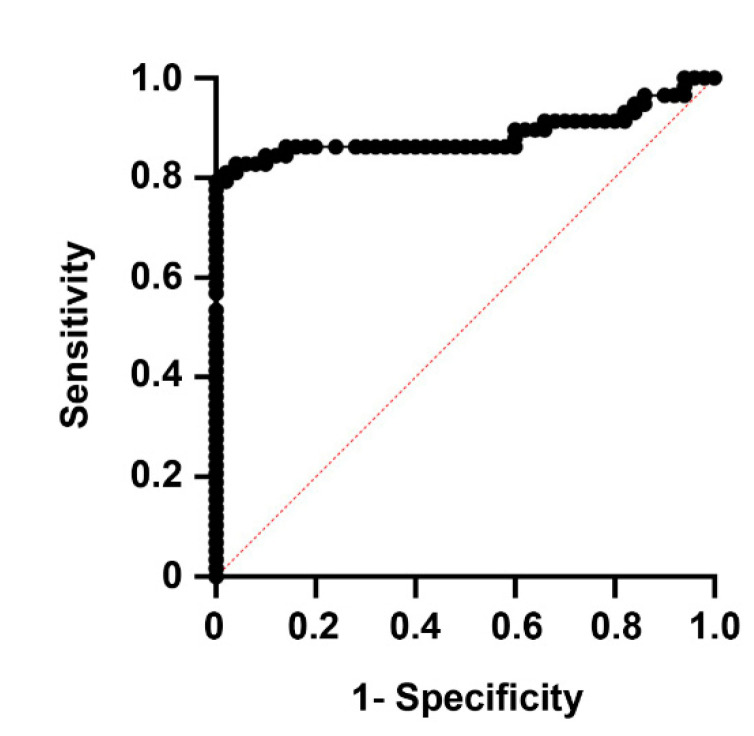
Receiver operating characteristic (ROC) curve showing the predictive power of AM levels on whether experimental subjects belong in the healthy or disease groups. The area under the curve (AUC) was 0.89, with 80.0% sensitivity and 100% specificity (*p* < 0.0001).

The optimal cutoff for AM to differentiate between stroke patients and healthy controls, according to Youden’s J statistic, was 69.0 pg/mL (J = 0.80). A receiver operating characteristic (ROC) curve using this cutoff value shows an area under the curve (AUC) of 0.89, with 80.0% sensitivity and 100% specificity (*p* < 0.0001) (Figure 2).

### 3.2. The Temporal Pattern of AM Levels Predicts Prognosis and Patient Dependency

**Figure 3:**
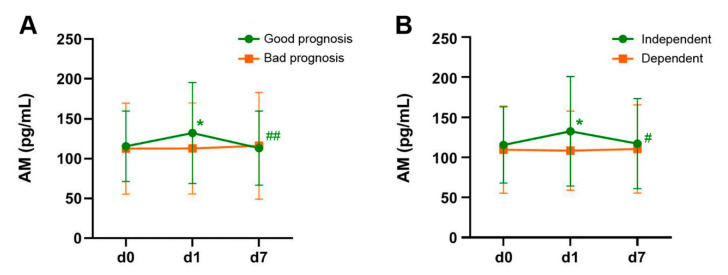
Evolution of AM levels through time in patients with favorable (green lines) or unfavorable (orange lines) outcome, as measured by the NIHSS (**A**) and the mRankin (**B**) scales. Patients with a positive evolution of the disease were characterized by an initial elevation of AM levels from d0 to d1 followed by a decline to initial levels by d7. Patients with unfavorable outcome had no significant changes in AM levels. Bars represent the mean and SD of all patients in the group. *: *p* < 0.05 vs. d0; #: *p* < 0.05 vs. d1; ##: *p* < 0.01 vs. d1.

Patients were classified, depending on their NIHSS at the time of their hospital discharge (d7), in two groups: good prognosis (NIHSS ≤ 3) and bad prognosis (NIHSS > 3). The AM levels remained constant in the bad prognosis group (*p* = 0.64) but there was a significant change in AM levels for the patients with good prognosis (*p* < 0.001). In these patients, there was a significant increase from d0 to d1 (*p* = 0.012), followed by a significant decrease from d1 to d7 (*p* = 0.006). There was no significant difference between AM levels at d0 and d7 (*p* = 0.63) (Figure 3A).

A similar pattern was observed when classifying patients depending on the mRankin scale, measured at 3 months after stroke. Patients whose mRankin ≤ 2 are considered independent whereas those with mRankin > 2 are considered dependent. In a similar fashion to the one observed with the NIHSS, there was no temporal changes in AM levels among dependent stroke patients (*p* = 0.79). On the other hand, patients that regained independency by 3 months had significant changes in AM levels (*p* = 0.006). In these patients, AM levels increased from d0 to d1 (*p* = 0.016) and decreased to basal levels from d1 to d7 (*p* = 0.03) (Figure 3B).

No association was found between AM levels and mortality (*p* = 0.59) or hematoma growth (*p* = 0.65).

### 3.3. NOx Levels Are Lower in Hemorrhagic Stroke Patients Than in Healthy Controls

**Figure 4:**
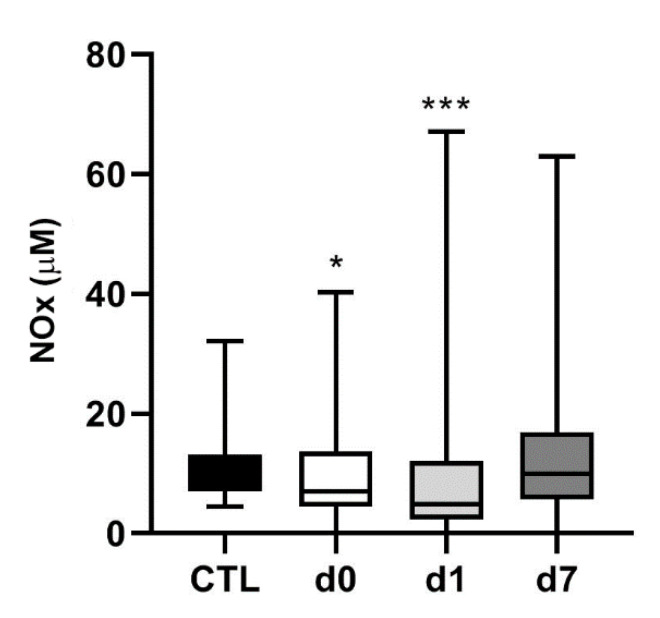
Circulating NOx levels in hemorrhagic stroke patients. NOx levels were measured in the same healthy volunteers (CTL) and hemorrhagic stroke patients as in Figure 1. In patients, blood samples were taken on admission (d0), 24 h later (d1), and at hospital discharge (d7). Box plots represent the interquartile range with the median as a horizontal line. Whiskers encompass the maximum and minimum values of the population. *: *p* < 0.05; ***: *p* < 0.001 vs. CTL.

Since AM and NO are intimately related in vascular biology [37], we also measured the levels of NOx in the same clinical samples. In healthy control subjects, NOx levels were 9.1 µM (7.1–12.1). In hemorrhagic stroke patients, NOx levels were lower on d0 (7.0 µM (4.6–12.9)) (*p* = 0.04) and d1 (4.8 µM (2.4–11.5)) (*p* < 0.001), and went back to normal on d7 (9.9 µM (5.9–16.3)) (Figure 4). A high level of variability was obtained among patients and that was probably responsible for the lack of association of these values with the main variables of the study.

## 4. Discussion

We have shown that acute intracerebral hemorrhage patients present higher levels of circulating AM when compared to the healthy population. Furthermore, those patients whose AM levels sharply increased from d0 to d1 and then went back to basal levels by d7 had more favorable outcomes as measured by NIHSS at d7 and mRankin at 3 months.

The study has some limitations. First, it was a single-center observational (cross-sectional) study and has a relatively small number of enrolled subjects. Larger and multicentric studies will be needed to corroborate our results. Second, due to the source of volunteers (Blood Bank donors), the healthy control group was not matched by age with the stroke population. Previous studies have shown that AM expression slightly increases with age, at least in the brain [38]. Nevertheless, we studied whether there was a correlation in our data between age and AM levels and we found no significant correlation, thus indicating that this is not a big issue.

All previous studies have shown elevated AM levels, at least at admission, both in ischemic [24,25,39,40] and hemorrhagic [26,27,28] stroke patients when compared to healthy controls. Therefore, our study confirms previous findings; in fact, our levels for both healthy controls and stroke patients at admission are almost identical to those published by Wang et al. [26]. The fact that AM levels increase after stroke is to be expected if we consider that the *Adm* gene promoter contains several hypoxia response elements (HRE) which would induce AM transactivation in the presence of the HIF-1 transcription factor, which is activated under hypoxia [17].

A previous study from our laboratory showed that, in ischemic stroke, circulating AM levels were higher than in the control population at admission and at d1, but returned to normal by d7 [25]. In contrast, our current observations in hemorrhagic stroke show that the AM levels at d7 were still significantly higher than those measured in healthy volunteers. This clear difference may be based on the higher severity of hemorrhagic stroke [41], which may require longer periods of time to bring down to normal the AM levels.

Furthermore, in ischemic stroke, higher levels of AM at d1 predicted increased neurological severity at 7 days and at 3 months [25], but our current data on hemorrhagic stroke indicate that those patients whose AM levels increased at d1 and then went down by d7 had more favorable outcomes as measured by the NIHSS and mRankin scales. This striking discrepancy indicates that both types of stroke are very different clinical entities and that AM may play different roles in their physiopathology. It seems that AM may be damaging for ischemic stroke whereas it is beneficial for hemorrhagic stroke. Despite the clear connection between AM and the cardiovascular system [18,37], understanding the exact contribution of AM to stroke resolution is not an easy task and the literature is full of apparent controversies, which may ultimately reflect the high complexity of the system [42]. For instance, therapeutic treatment of animal models of ischemic stroke with AM peptide may result in either damaging [43] or protective [44,45,46] effects. In another example, from our own laboratory, we described that genetically eliminating the *adm* gene from neurons increased stroke volume in a mouse model of middle cerebral artery occlusion [23]; but when we eliminated the same gene from endothelial cells, we obtained the reverse result [47]. To date, no AM levels have been studied in animal models of hemorrhagic stroke. Those studies may offer additional clues on the specific role of AM in the pathophysiology of this condition.

Interestingly, the AM levels taken at any time during the first week following hemorrhagic stroke can be used as a potential diagnostic biomarker for hemorrhagic stroke, with high sensitivity and specificity. If a simple and fast device could be designed to rapidly measure blood AM levels, it could be useful for the quick triage of patients when in doubt of their diagnosis [13,14]. Furthermore, knowing the AM levels at d1 may offer a useful prognostic biomarker for hemorrhagic stroke patients.

Several modulators of AM physiology have been described, and some of them could be used as novel therapies for hemorrhagic stroke once the exact contribution of AM is established. These modulators include monoclonal antibodies [48], polyclonal antibodies against either the peptide [49,50] or the receptors [51], the peptide fragment AM22−52 [52], and small interfering RNAs [53]. In addition, several small molecules have been identified which can either increase or decrease AM functions [54]. It would be interesting to test whether these pharmacological modulators, specially the small molecules activating AM activity, contribute to hemorrhagic stroke resolution in animal models.

In previous studies, the circulating levels of NOx have been determined in ischemic [25,55] and hemorrhagic [56] stroke. In those studies, an initial decrease on NOx was observed compared to healthy volunteers, which progressively increased through the hospital stay. Furthermore, NOx levels correlated with clinical outcomes. Thus, our data confirmed previous studies regarding the time evolution of NOx levels but we did not obtain any association with clinical outcomes, probably due to the high variability found among our subjects. NO is supposed to exert a beneficial effect on stroke patients and NO inhalation has been shown to reduce brain damage and mortality associated to hemorrhagic stroke [57]. These benefits seem to be related to the endothelial form of the NOS enzyme, since abrogation of this protein results in worse outcomes for genetically modified mice [58].

## 5. Conclusions

In conclusion, we have shown that circulating AM levels increase after suffering acute hemorrhagic stroke and that those levels change only in patients with favorable outcomes measured at 7 days and 3 months post-stroke. Therefore, AM levels may constitute a diagnostic and prognostic biomarker for this disease, indicating that AM is a positive mediator of stroke recovery, opening the door for new treatments based on AM modulation.

## Figures and Tables

**Table 1:** Clinical characteristics of the patients and volunteers included in the study.

		Hemorrhagic Patients	Healthy Volunteers	*p* Value
**Age** [years], median (Q1–Q3)		81 (72.2–87)	46.5 (40–54)	***<0.001***
**Sex** [males], (%)		38 (59.4%)	31 (62.0%)	0.91
**Risk factors**				
	Arterial hypertension	49 (76.6%)		
	Diabetes mellitus	15 (23.4%)		
	Dyslipidemia	20 (21.9%)		
	Atrial fibrillation	18 (28.1%)		
	Stroke	16 (25.0%)		
	Ischemic heart disease	8 (12.5%)		
	Cognitive impairment	8 (31.2%)		
	Smoking	17 (26. 6%)		
**Previous treatment**				
	Antihypertensives	45 (70.3%)		
	Anti-aggregants	19 (29.7%)		
	Anticoagulants	20 (31.8%)		
	Statins	21 (32.8%)		
**Hematoma localization**				
	Supratentorial	41 (64.1%)		
	Infratentorial	6 (9.4%)		
	Lobar	14 (21.8%)		
	Mixed	3 (4.7%)		
**Rankin scale**				
Basal	0–1–2	54 (84.4%)		
	3–4	10 (15.6%)		
	5–6	0 (0.0%)		
3 months	0–1–2	28 (43.7%)		
	3–4	12 (18.7%)		
	5–6	24 (37.5%)		
**NIHSS**, median (Q1–Q3)				
Basal		7.5 (2–16)		
Hospital discharge		5 (2–6.7)		
**Hematoma** at d0 (cm^3^)		4.5 (1–13.9)		
**Hematoma** at d1 (cm^3^)		4.6 (1–15.0)		
AM at d0 (pg/mL), median (Q1–Q3)		105.8 (74.6–157.8)	47.7 (43.3–52.8)	***<0.0001***
AM at d1 (pg/mL), median (Q1–Q3)		106.4 (79.3–166.4)		
AM at d7 (pg/mL), median (Q1–Q3)		109.7 (79.2–154.4)		
NOx at d0 (µM), median (Q1–Q3)		7.0 (4.6–12.9)	9.1 (7.1–12.1)	***0.04***
NOx at d1 (µM), median (Q1–Q3)		4.8 (2.4–11.5)		
NOx at d7 (µM), median (Q1–Q3)		9.9 (5.9–16.3)		

## Data Availability

The data presented in this study are available on request from the corresponding author.
